# Identification and Heterologous Expression of the Biosynthetic Gene Cluster Encoding the Lasso Peptide Humidimycin, a Caspofungin Activity Potentiator

**DOI:** 10.3390/antibiotics9020067

**Published:** 2020-02-07

**Authors:** Marina Sánchez-Hidalgo, Jesús Martín, Olga Genilloud

**Affiliations:** Fundación MEDINA, Avenida del Conocimiento 34, 18016 Granada, Spain; jesus.martin@medinaandalucia.es (J.M.); olga.genilloud@medinaandalucia.es (O.G.)

**Keywords:** Humidimycin, MDN-0010, lasso peptide, RiPP, *Streptomyces humidus*, genome mining, biosynthetic gene cluster, Gibson assembly, heterologous expression

## Abstract

Humidimycin (MDN-0010) is a ribosomally synthesized and post-translationally modified peptide (RiPP) belonging to class I lasso peptides, and is structurally related to siamycins, which have been shown to have strong antimicrobial activities against Gram-positive bacteria and to possess anti-HIV activity. Humidimycin was isolated from the strain *Streptomyces humidus* CA-100629, and was shown to synergize the activity of the fungal cell wall inhibitor caspofungin. In this work, the biosynthetic gene cluster of humidimycin was identified by genome mining of *S. humidus* CA-100629, cloned by Gibson assembly, and heterologously expressed.

## 1. Introduction

Natural products are non-essential small molecules produced by plants, microbes, and invertebrates. More than 70% of the natural product scaffolds used to produce clinically relevant anti-infective molecules are produced by Actinobacteria [1,2,3]. Advances in next-generation sequencing (NGS) technologies and genome mining approaches have reinvigorated the search for novel bioactive natural products [4,5]. The continuous development of bioinformatics tools and databases for the automated scanning and annotation of secondary metabolite gene clusters, such as antiSMASH (antibiotics and secondary metabolites analysis shell) [6], MIBiG (minimum information about a biosynthetic gene cluster) [7], or PRISM (prediction informatics for secondary metabolomes) [8], is enabling the discovery of new biosynthetic gene clusters (BGCs), including those encoding ribosomally synthesized and post-translationally modified peptides (RiPPs), which have been shown to be more widespread than first estimated [9,10].

**Figure 1:**
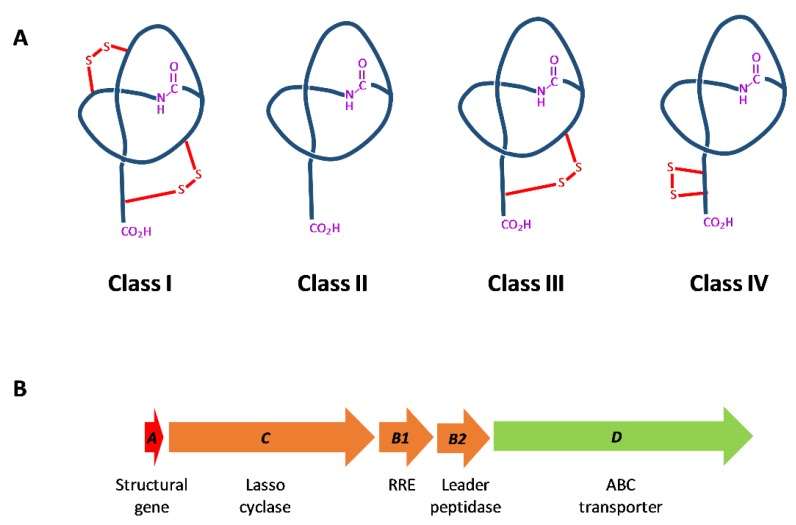
Classification of lasso peptides according to their different topologies (**A**) and general overview of lasso peptide biosynthetic genes (**B**).

Among RiPPs, lasso peptides are cyclic peptides, containing an N-terminal macrolactam ring comprised of seven to nine amino acid residues and a linear C-terminal peptide tail threaded through the ring. Lasso peptides are classified into four classes, depending on the number of disulfide bonds present (Figure 1A) [11]: Class I lasso peptides, only produced by strains of *Streptomyces*, have two disulfide bridges in the molecule; class II lasso peptides have no disulfide bonds; and class III have only one disulfide bond. Tietz et al. [12] described the new class IV to include the topology of lasso peptide LP2006, which contains two Cys forming a disulfide bridge on the tail and not linking the tail to the macrocycle, as occurs in class I and III lasso peptides.

In recent years, genome mining approaches have considerably increased the number of identified lasso peptides and other RiPPs BGCs, and contributed to a better understanding of their biosynthesis [9,13]. In addition to antiSMAH and PRISM, platforms like BAGEL (bacteriocin genome mining tool) [14], RiPPER (RiPP precursor peptide enhanced recognition) [15], RiPPMiner (ribosomally synthesized post-translationally modified peptides miner) [16], and RODEO (rapid ORF description & evaluation online) [17] can efficiently mine genomic sequence data to search for lasso peptides.

Lasso peptides are synthesized from a precursor peptide, named A protein, which is processed by a leader peptidase (the B protein), homologous to a transglutaminase, and by a lasso cyclase (C-protein), homologous to an asparagine synthase, which is involved in the formation of the 7-9 residue macrolactam ring [12] (Figure 1B). Some lasso peptide gene clusters present B1 gene encoding for the N-terminal domain of the B protein and B2 gene encoding for the C-terminal domain. The B1 protein, also described as E-protein [12], contains the RiPP recognition element (RRE) [18], which recognizes the leader peptide and directs enzymatic modification [19]. Then, the B2 protein cleaves the leader peptide. The structural basis of leader peptide recognition by these so-called “split-B” proteins have been characterized in depth in Actinobacteria and Firmicutes [20]. 

Other genes present in many lasso peptide gene clusters encode for an ABC-transporter, presumably involved in the secretion and the self-immunity of the lasso peptide [21]. In other cases, a gene encoding for an isopeptidase is present instead of the ABC transporter. It has been proposed that clusters containing ABC-transporters might produce antimicrobial lasso peptides, while those containing isopeptidases might produce lasso peptides with another function [21]. Lasso peptide gene clusters are often flanked by regulatory proteins, but these have been rarely studied [13].

Humidimycin is a class I lasso peptide that was previously discovered by our research team as a new synergist of the of the fungal cell wall biosynthesis inhibitor caspofungin (CAS) [22]. The dramatic increase in the number of cases of life-threatening fungal infections and the lack of effective drugs make the development of novel approaches to fight against these infectious diseases necessary. The combination of humidimycin and CAS strongly increases the CAS efficacy against *Aspergillus fumigatus* and *Candida albicans*. The analysis of transcriptomes and selected *A. fumigatus* mutants has suggested that humidimycin affects the high osmolarity glycerol (HOG) response pathway and hits the CAS salvage pathway in human pathogenic fungi, a target that can be used to potentiate CAS activity [22]. Since humidimycin has no antifungal activity on its own and lacks in vitro cytotoxicity, this molecule could be an excellent example to propose the development of new combined treatments of invasive fungal infections [22].

Humidimycin was isolated from liquid culture broths of the actinomycete *Streptomyces humidus* CA-100629, and its structural elucidation showed that it is closely related to siamycins, which have anti-HIV activity and strong antimicrobial activities against Gram-positive bacteria [23,24,25,26]. The structure of humidimycin, a 21-amino-acid peptide that is cyclized from the side chain of Asp9 to the N-terminus of Cysl, has the topology of class I lasso peptides. Two disulfide bonds containing linkages Cys1 ➔ Cys13 and Cys7 ➔ Cys19 form the tricyclic structure [22]. Recently, Tan et al. [27] have found that siamycin I binds and inhibits the action of lipid II, the essential precursor molecule involved in peptidoglycan formation. Given the few differences in the amino acid sequences of class I lasso peptides, it might be also expected that other molecules of this group, including humidimycin, also inhibit peptidoglycan biosynthesis [27].

The BGCs of additional class I siamycin-like molecules (aborycin, specialicin, and MS-271) have been recently identified by genome mining, and some of them have been heterologously expressed [28,29,30]. 

In this work, we present the identification, cloning, and heterologous expression of the humidimycin BGC (*hum*) after genome mining of the producer strain *S. humidus* CA-100629. We also demonstrate that the C-terminal tryptophan of humidimycin is epimerized in both wild-type and heterologous strains.

## 2. Results and Discussion

### 2.1. Identification and Analysis of Humidimycin Biosynthetic Gene Cluster

**Figure 2:**
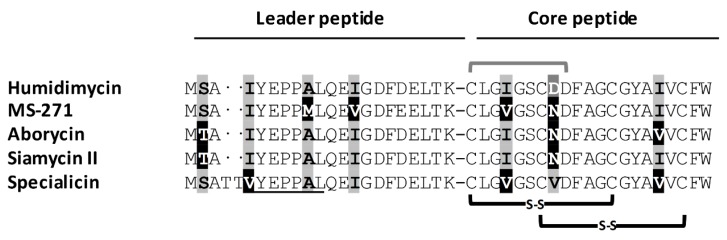
Comparison of the leader and core sequences of the siamycin-like pre-propeptides. The disulfide bonds are indicated in black, and the amide bond between the N-terminal Cys and the side chain of the aspartic residue at position 9 is indicated in grey. Underlined residues correspond to the YxxPxL motif recognized by the RiPP recognition element (RRE)-containing protein. Differences in the sequences are highlighted.

The humidimycin BGC (*hum*) was located by mining the genome of strain CA-100629 (data not shown). The core sequence of humidimycin (CLGIGSCDDFAGCGYAIVCFW) was used to query the genome sequence in a tblastn and allowed us to find an open reading frame (ORF) (*humA*) that unambiguously corresponds to the structural gene. Among the described siamycin-like peptides, humidimycin is the only one having an aspartic residue in position 8 (Figure 2). From the nucleotide sequence of *humA*, we were able to deduce the amino acid sequence of the leader peptide of humidimycin (MSAIYEPPALQEIGDFDELTK). As shown in Figure 2, the leader peptide is very similar to the leader peptides of MS-271, aborycin, siamycin II, and specialicin, and contains the conserved YxxPxL motif and a threonine residue that are required for leader peptide recognition by the RRE in Actinobacteria [11,12].

**Figure 3:**
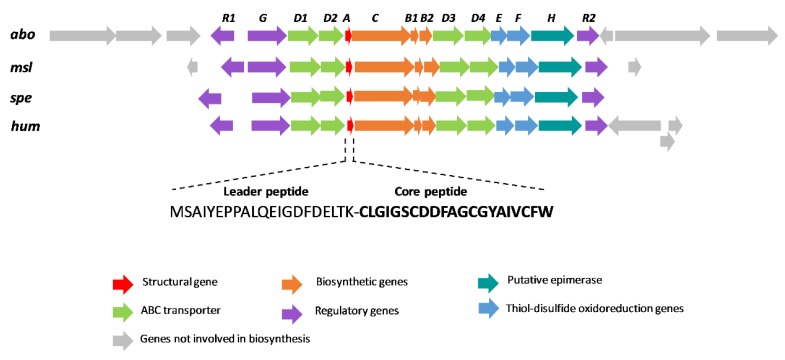
Schematic representation of the biosynthetic gene cluster (BGC) of siamycin-like lasso peptides (*abo*: aborycin BGC from *Streptomyces* sp. SCSIO ZS0098; *msl*: MS-271 BGC from *Streptomyces* sp. M-271; *spe*: specialicin BGC from *S. specialis* JCM 16611T; *hum*: humidimycin BGC from *S. humidus* CA-100629). The sequences of the leader and core peptides of humidimycin are shown.

The analysis of the ORFs present in a region of ~15 Kb up- and downstream the structural gene indicated that this cluster (*hum*) has a gene organization similar to those of other siamycin-like peptides (Figure 3). In fact, the proteins encoded in the *hum* BGC share a high degree of similarity with those described in other class I lasso peptides, which has permitted the proposal of a function for each gene based on these homologies [29,30] (Table 1). The *hum* cluster encodes a precursor peptide (HumA) that is processed by a split B protein HumB1 containing an RRE that joins the pre-propeptide and prefolds it *via* ATP-driven hydrolysis. Then, HumB2 cleaves the leader peptide to generate a 21-amino-acid linear peptide. Next, the lasso peptide cyclase HumC forms a macrolactam ring between the amino group of N-terminal Cys1 and the carboxyl group of Asp9, and the oxidoreductases HumE and HumF establish disulfide bonds between Cys1 and Cys13 and between Cys7 and Cys19, respectively. Finally, proteins HumD1/D2/D3/D4 should be involved in the export the peptide. The cluster also has one histidine kinase (HumG) and two DNA-binding response regulators (HumR1/R2) that are most probably involved in the regulation of gene expression. 

Feng et al. [30] demonstrated the involvement of the homologous regulators MslR1, MslG, and MslR2 in the production of MS-271. They independently cloned *mslR1*, *mslG*, and *mslR2* genes under the control of an *erm*EP* promoter and determined that MslR2 and MslG significantly increased product yield, while MslR1 decreased the production of MS-271. Given the high degree of homology of *hum* and *msl* regulators (Table 1), the production of humidimycin might follow the same regulation.

The cluster also harbors a gene encoding HumH, a protein homologous to MslH and SpeH, which have been proposed to epimerize Trp21 in MS-271 and specialicin, respectively [29,30]. Since *spe*, *msl*, and *hum* clusters lack an obvious epimerase, and the H protein is the only one of unknown function, is likely to be responsible for the epimerization of the C-terminal Trp of humidimycin [31]. The deletion of the *mslH* gene completely abolished MS-271 production [30], a fact that demonstrated that MslH is required for MS-271 biosynthesis, as most probably is HumH. Recently, Ogasawara et al. [32] described that PgsA, which has a 52% identity to MslH, catalyzes the epimerization of L-Glu residues to D-Glu in the growing poly-γ-glutamic acid (PGA) chain, a polymer that is present in the mucilage of many bacteria and which contains D- and L-Glu connected *via* an amide bond. The authors propose that MslH would act in a similar manner to PgsA in MS-271 biosynthesis, although the detailed reaction mechanism is still unclear.

### 2.2. Cloning and Heterologous Expression of Humidimycin Biosynthetic Gene Cluster

To prove that the *hum* cluster is responsible for humidimycin production, and to check if the C-terminal Trp of humidimycin is epimerized, the 14,987 bp fragment containing the *hum* genes was cloned in pCAP01 by Gibson assembly, yielding the plasmid pHUM. To that end, four overlapping fragments of 4.590 Kb, 4.452 Kb, 3.070 Kb, and 3.063 Kb were amplified by PCR. A XhoI-linearized vector pCAP01 (9 Kb) was also amplified. The five fragments were combined for Gibson assembly, and the reaction was transformed into *E. coli* NEB 10-beta competent cells. Clones were checked by restriction analysis, and one of the clones harboring pHUM was selected to purify the vector. 

Then, pHUM was used to transform Cm^R^ *E. coli* ET12567. Since pHUM contains the kanamycin-resistant marker, we could not directly transform non-methylating Cm^R^ Km^R^ *E. coli* ET12567/pUB307. Thus, we performed three independent, triparental, intergeneric conjugations, using *E. coli* ET12567/pHUM and ET12567/pUB307 as donor strains and the spores of each of the heterologous hosts (*S. coelicolor* M1152, M1154 and *S. albus* J1074) as recipient strains. For the negative controls, a triparental conjugation was also made, using *E. coli* ET12567/pCAP01 and ET12567/pUB307 as donor strains with the same recipient strains. Transconjugants were checked by PCR with primers HumidA_fw and HumidA_rv to confirm the integration of the *hum* BGC into the chromosomes of the heterologous hosts.

Five positive transconjugants from each heterologous host, together with the negative controls, were grown on liquid RAM2, the original humidimycin-producing medium, over 7 days at 28 °C, and then acetone extracts from the cultures were obtained. After removing of the solvent, the residue was resuspended in 20% dimethyl sulfoxide (DMSO)/water and analyzed by LC-HRESI-TOF.

**Figure 4:**
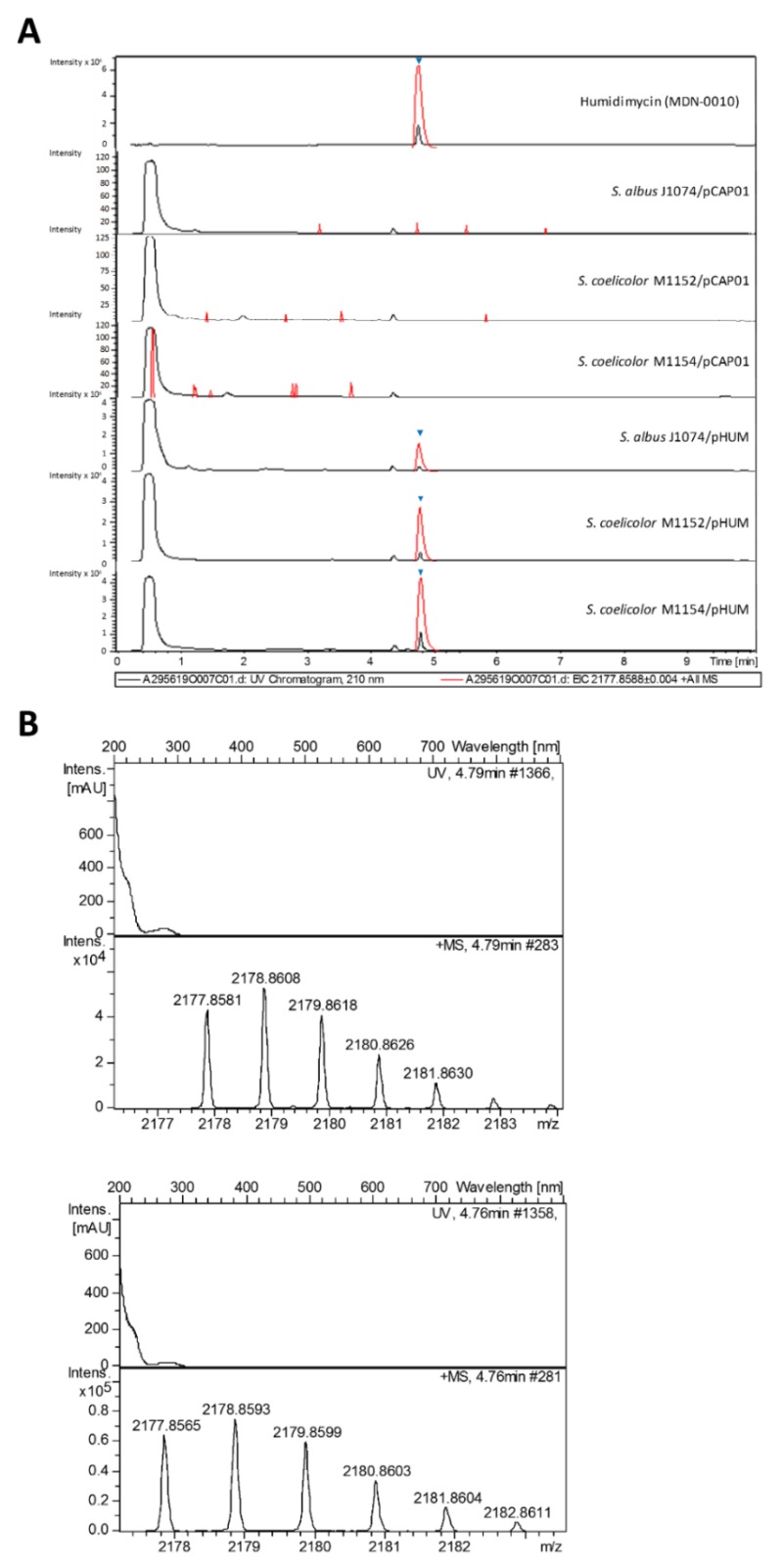
UV profiles at 210 nm (black) and extracted ion chromatograms (red) (**A**) at 2177.8588 ± 0.004 (C_98_H_133_N_22_O_27_S_4_^+^) of humidimycin, the negative controls *S. albus* J1074/pCAP01, *S. coelicolor* M1152/pCAP01, and *S. coelicolor* M1154/pCAP01, and the transconjugants *S. albus* J1074/pHUM, *S. coelicolor* M1152/pHUM, and *S. coelicolor* M1154/pHUM. ▼: humidimycin. (**B**) Experimental UV and mass spectra of M + H^+^ humidimycin adduct (C_98_H_133_N_22_O_27_S_4_^+^) from *S. coelicolor* M1154/pHUM (top), and from the original producer strain CA-100629 (bottom).

All the transconjugants showed a peak at 4.8 minutes, which was absent in the negative controls (Figure 4A) and which coincided with the retention time of elution of the humidimycin standard. The perfect correlation between the UV spectrum, exact mass, and isotopic distribution between the humidimycin standard (Figure 4B, top) and the component isolated from the transconjugant *S. coelicolor* M1154/pHUM (Figure 4B, bottom) unequivocally confirmed that the latter corresponded to humidimycin. *S. albus* J1074 and the engineered strains *S. coelicolor* M1152 and *S. coelicolor* M1154 have been largely used as heterologous hosts to produce of a broad range of compound classes with improved yields [33]. In most cases, *S. albus* J1074 provides better yields than the *S. coelicolor* strains, a fact that could be due to the presence of an additional active vector integration *attB* site in its chromosome [34]. However, in the case of pHUM transconjugants, the production of humidimycin was higher in the transconjugant *S. coelicolor* M1154-pHUM (Figure 4A).

### 2.3. Marfey’s Analysis of Humidimycin C-terminal Tryptophan

**Figure 5:**
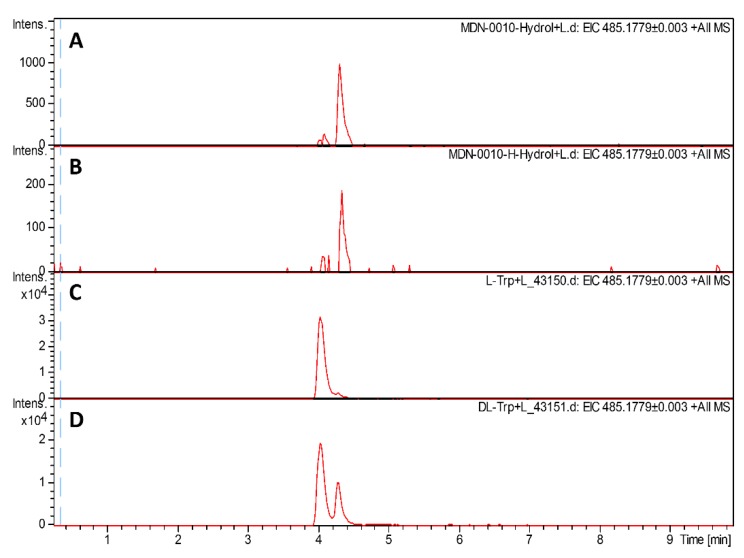
Marfey’s chiral analysis of the C-terminal tryptophan (Trp) using 1-fluoro-2,4-dinitrophenyl-5-L-valine-amide (L-FDVA). The LC-MS profile was monitored at 485.1779 *m*/*z* (M + H^+^ of FDVA derivatives of Trp). (**A**) Humidimycin from strain *S. humidus* CA-100629, (**B**) humidimycin from *S. coelicolor* M1154/pHUM cultures, (**C**) L-Trp standard. and (**D**) L-Trp + D-Trp standard.

When the structure of humidimycin was determined [22], all the amino acids were assumed to have an L-configuration analogous to what had been described for siamycin II [35]. However, considering the similarity of the biosynthesis with that of MS-271 [30] and specialicin [29], which have been described as having a D-tryptophan, it was reasonable to think that humidimycin, and by extension the rest of siamycins, could also contain a D-Trp. We determined the absolute configuration of the C-terminal Trp using Marfey’s method, and confirmed the D-stereochemistry of the C-terminal Trp of humidimycin isolated from both the original producer strain and the transconjugant *S. coelicolor* M1154/pHUM (Figure 5). As the *hum* cluster lacks obvious epimerase genes to catalyze the stereochemical inversion of Trp21, and (as stated above) HumH is homologous to SpeH and MslH, we propose this protein to also be responsible for the epimerization.

### 2.4. Genome Mining of Additional Humidimycin-like Biosynthetic Gene Clusters

**Figure 6:**
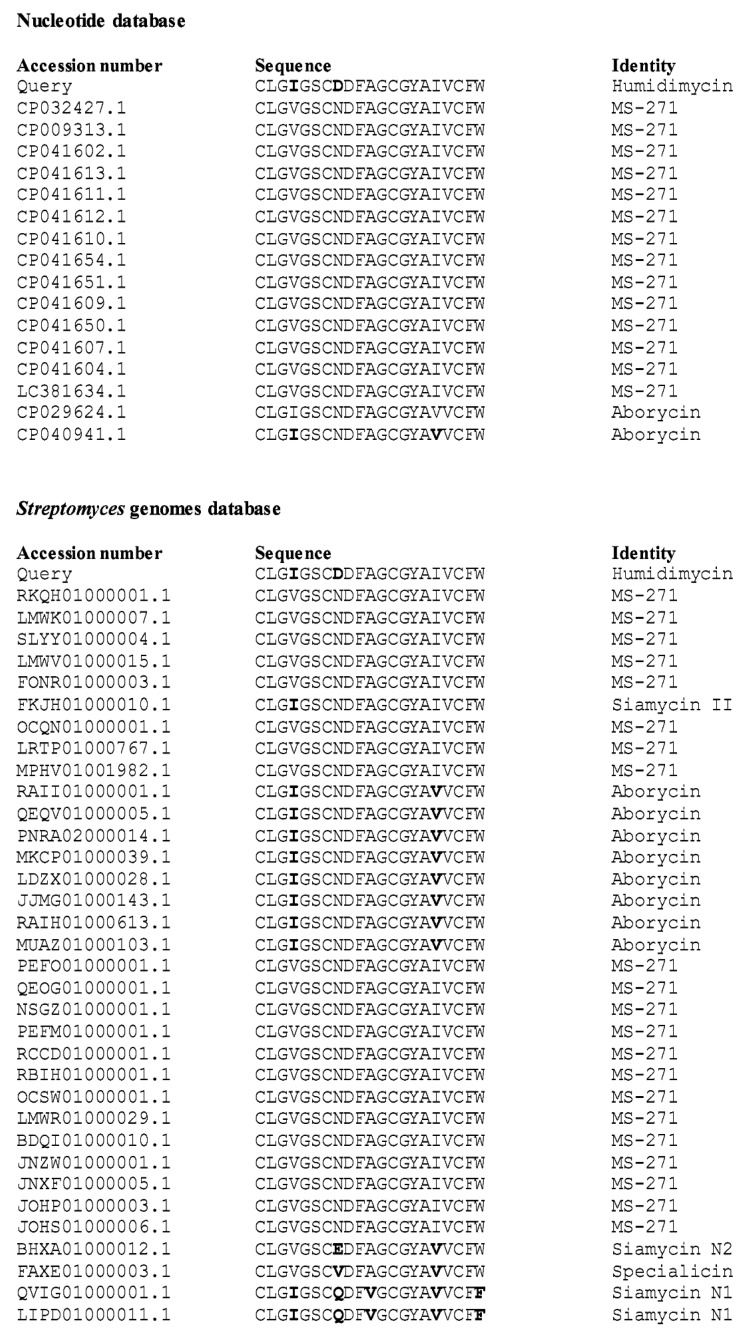
Humidimycin homologs found by BLAST against the nucleotide and *Streptomyces* WGS databases from NCBI. Letters in bold indicate amino acid differences.

**Figure 7:**
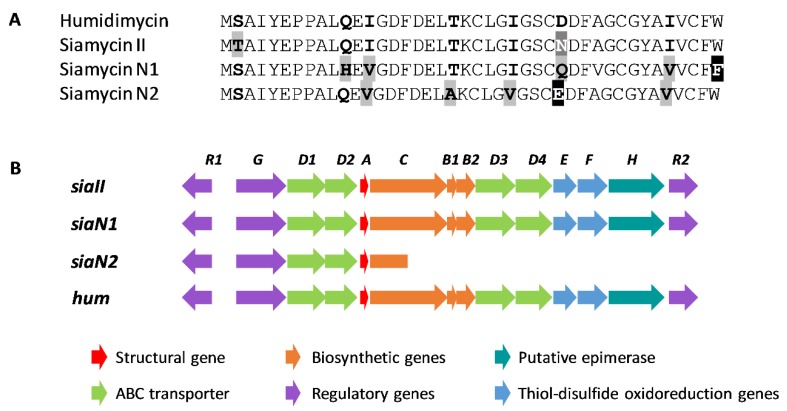
Comparison of the pre-propeptide sequences (**A**) and the biosynthetic gene cluster (BGC) (**B**) of humidimycin, siamycin II, and the new siamycins (N1 and N2) identified by genome mining.

A tblastn search of the humidimycin core peptide against both nucleotide and *Streptomyces* whole genome sequences (WGS) databases from NCBI has shown that no other humidimycin gene clusters are contained in these genomes, and that siamycin I/MS-271 and siamycin IIII/RP 71955/aborycin are the siamycin-like lasso peptides most frequently detected (Figure 6). We also found an undescribed BGC for siamycin II in the genome of the strain *Streptomyces* sp. F-7 (GenBank FKJH01000010.1) that shares a high degree of homology with the rest of siamycin-like clusters (Figure 7B).

This search also allowed us to find two new siamycin-like peptides (Figure 6 and Figure 7A); one, named for clarity as siamycin N1, in the genomes of *Streptomyces* sp. CB02056 (GenBank LIPD01000011.1) and *Streptomyces xanthocidicus* MMS17-GH009 (GenBank QVIG01000001.1), and another, named siamycin N2, in the genome of *Streptomyces* sp. NL15-2K (GenBank BHXA01000012.1). In the case of siamycin N2, the pathway lacks the genes necessary to synthesize a lasso peptide, and contains a truncated C protein. However, in the case of siamycin N1, a complete and typical siamycin cluster was found in both genomes (Figure 7B). It is interesting to note in the case of siamycin N1 that the residue in position 21 is a phenylalanine and not a tryptophan, as has been described for all the siamycin-like peptides (Figure 7A). Considering that the siamycin N1 BGC also harbors a HumH homolog, it would be of great interest to study whether this gene is also able to epimerize Phe21. The study of the biological activities of siamycin N1 would pave the way to the detailed molecular characterization of this group of molecules.

## 3. Materials and Methods 

### 3.1. Bacterial Strains and Plasmids

Strain *Streptomyces humidus* CA-100629 was isolated from a soil collected in Almería (Spain) and deposited in MEDINA culture collection [22]. NEB 10-beta competent *E. coli* (New England BioLabs, Ipswich, MA, United States), *E. coli* ET12567 (LGC Standards, Manchester, NH, United States), and *E. coli* ET12567/pUB307 [36] (kindly provided by José Antonio Salas) were employed throughout the cloning and conjugation processes. Vector pCAP01, a *S. cerevisiae/E. coli*/actinobacteria shuttle vector allowing site-specific φC31 integration of the cloned gene cluster into the chromosomes of heterologous Actinobacteria hosts [37], was a gift from Bradley Moore (Addgene plasmid #59981; http://n2t.net/addgene:59981; RRID: Addgene_59981). *Streptomyces coelicolor* M1152 and M1154 heterologous hosts [38] were kindly gifted by Mervyn Bibb. The *Streptomyces albus* J1074 heterologous host [39] was generously provided by José Antonio Salas.

### 3.2. Growth and Culture Conditions

*Streptomyces humidus* CA-100629 was cultured on ATCC-2 liquid medium (soluble starch 20 g/L, glucose 10 g/L, NZ Amine Type E 5 g/L, meat extract 3 g/L, peptone 5 g/L, yeast extract 5 g/L, calcium carbonate 1 g/L; pH 7) and grown in an orbital shaker at 28 °C, 220 rpm, and 70% relative humidity. *E. coli* strains were routinely cultured in LB broth (Miller) (Merck KGaA, Darmstadt, Germany) (37 °C, 250 rpm) and Difco LB agar Lennox (Fisher Scientific, Madrid, Spain) (37 °C, static). Intergeneric conjugations were carried out on MA (MOPS 21 g/L, glucose 5 g/L, yeast extract 0.5 g/L, beef extract 0.5 g/L, casamino acids 1 g/L, agar 25 g/L; pH 7). Transconjugants were grown on MA. Antibiotics were supplemented when required for selection of transformants at the following final concentrations: kanamycin (50 μg/mL), nalidixic acid (25 μg/mL), and choramphenicol (25 μg/mL). For heterologous expression experiments, RAM2 medium was used (corn meal 4 g/L, glucose 10 g/L, maltose 15 g/L, pharmamedia 7.5 g/L, primary yeast 5 g/L; pH 7), and the recombinant strains were incubated for 7 days on an orbital shaker at 28 °C, 220 rpm, and 70% relative humidity.

### 3.3. General Molecular Biology Techniques

Restriction endonucleases and Q5 High-Fidelity polymerase were purchased from New England Biolabs (Ipswich, MA, United States). Calf intestinal alkaline phosphatase (CIAP) was purchased from Invitrogen (Waltham, MA, United States). Gibson Assembly® Ultra Kit was purchased from VWR (Chester, PA, United States). All the primers employed were purchased at Sigma-Aldrich (St. Louis, MO, United States) and are described in Table 2. A QIAprep Spin Miniprep Kit (Qiagen, Hilden, Germany) was employed for the purification of plasmid DNA, and the Illustra^TM^ GFX^TM^ PCR DNA and Gel Band Purification Kit (GE Healthcare, Boston, MA, United States) was used for the purification of DNA amplicons from agarose gels and enzymatic reactions.

### 3.4. Location and Analysis of Humidimycin Biosynthetic Gene Cluster

The sequence of the humidimycin BGC has been deposited at GenBank under the accession number MN956991. To identify the structural gene and the rest of ORFs present in the cluster, and to assign functions, the tBlastn and blastp algorithms were used (https://blast.ncbi.nlm.nih.gov), as well as antiSMASH [6].

### 3.5. Gibson Assembly Cloning of Humidimycin Biosynthetic Gene Cluster

Cloning of humidimycin BGC was performed by Gibson assembly. Four overlapping fragments between 3 and 4.5 Kb were PCR amplified using the genomic DNA of strain CA-100629 and the primers listed in Table 2. Also, vector pCAP01 was linearized with XhoI, and then used as the template in a PCR with primers pCAPHumGibF and pCAPHumGibR (Table 2), which have 5’ overhang sequences that would allow for Gibson assembly with the ends of the *hum* BGC. The PCR was digested with DpnI to eliminate the template DNA.

All the PCRs were purified after agarose gel electrophoresis using the Illustra^TM^ GFX^TM^ PCR DNA and Gel Band Purification Kit, and employed for a Gibson assembly reaction according to the manufacturer’s protocol. *E. coli* NEB 10-beta was transformed with the reaction, and kanamycin was used as a selection marker to screen for cells carrying the target plasmid.

Plasmid DNA was isolated from the transformants, and the correct cloning of the *hum* BGC was checked by restriction enzyme digestion and PCR amplification with primers HumidA_fw and HumidA_rv (Table 2).

### 3.6. Intergeneric Conjugation

Plasmid pHUM was introduced into *Streptomyces* hosts by triparental conjugation, as previously described [40]. In summary, purified pHUM was used to electroporate the methylation-deficient strain *E. coli* ET12567. Cells from *E. coli* ET12567/pUB307 and *E. coli* ET12567/pHUM were collected at an O.D. of 0.4-0.6, washed twice with LB to remove the antibiotics, resuspended in 100 µL of LB, and mixed with 50 µl of freshly activated spores of *S. coelicolor* M1152, M1154, and *S. albus* J1074. The mixtures were plated on MA and overlaid after about 16 h with nalidixic acid (25 μg/mL) and kanamycin (50 μg/mL). After few days of incubation, five colonies from each *Streptomyces* heterologous host were picked and streaked on MA plates containing nalidixic acid (25 μg/mL) and kanamycin (50 μg/mL). The insertion of pHUM into the *Streptomyces* hosts chromosomes was checked by PCR, employing the genomic DNA of the transconjugants and the primers HumidA_fw and HumidA_rv (Table 2).

### 3.7. Heterologous Expression of Humidimycin Biosynthetic Gene Cluster and LC-ESI-TOF Analysis

Seed cultures on ATCC-2 of the recombinant strains *S. coelicolor* M1152/pHUM, M1154/pHUM and *S. albus* J1074/pHUM, together with the corresponding negative controls harboring empty pCAP01 vector, were used to inoculate EPA vials containing 10 mL of RAM2 fermentation medium. The vials were incubated at 28 °C for 7 days at 220 rpm and 70% humidity, and then the broths were subjected to extraction with acetone. The organic solvent was evaporated to dryness and the extract resuspended to a final ratio of 20% DMSO/water. The microbial extracts were filtered and analyzed employing a Bruker maXis QTOF high resolution mass spectrometer coupled to a HPLC system, as previously described [41]. 

### 3.8. Marfey’s Analysis of Tryptophan

Two samples of humidimycin from strain CA-100629 (0.1 mg) and from *S. coelicolor* M1154/pHUM were treated with 6 N HCl containing a 5% of thioglicolic acid in a sealed vial at 110 °C for 24 h. The solutions were concentrated to dryness *in vacuo*. The hydrolysates were reconstituted in H_2_O (100 μL) and treated with a solution of 1-fluoro-2,4-dinitrophenyl-5-L-valine-amide (L-FDVA, 300 μL, 1% in acetone) and a 1 M solution of NaHCO_3_ (50 μL) in a sealed vial at 40 °C for 1 h. The reaction mixtures were neutralized with 1 N HCl (50 μL), and an aliquot (10 μL) was diluted with CH_3_CN (40 μL). The resulting solutions were analyzed by LC-MS employing a Zorbax SB-C8 column (2.1 × 30 mm, 3.5 μm) and a gradient elution profile of 10% B (90% CH_3_CN, 10% H2O, 0.01% TFA, 1.3 mM ammonium formiate)/90% A (10% CH_3_CN, 90% H2O, 0.01% TFA, 1.3 mM ammonium formiate) to 100% B over 6 min at a flow rate of 0.3 mL/min.

## 4. Conclusions

In this work, we have cloned and heterologously expressed in three different hosts the biosynthetic gene cluster of humidimycin (MDN-0010) from *Streptomyces humidus* CA-100629. The cluster is highly homologous to those of other siamycin-like peptides (aborycin, specialicin, MS-271), and contains genes encoding the precursor peptide (HumA), the enzymes involved in post-translational modifications (HumB1, HumB2, HumC, HumE, HumF), transporters (HumD1-D4), regulators (HumR1-R2, HumG), and a protein (HumH) that have been postulated to catalyze the epimerization of the tryptophan in position 21. We have in fact confirmed that the stereochemistry of Trp21 is D-, a modification that could increase the stability of the protein.

A search of novel siamycins in the NCBI microbial genomes database showed that new siamycin-like peptides are yet to be discovered. The few differences in the amino acid sequences of class I lasso peptides suggest that, as has been demonstrated with siamycin I and humidimycin, these molecules could also inhibit peptidoglycan biosynthesis and/or potentiate the activity of the antifugal compound caspofungin. The increasing number of sequenced genomes will help to better characterize of this group of molecules. 

## Figures and Tables

**Table 1:** Proposed functions and homologies of the proteins encoded in the *hum* cluster.

Proteins from *hum* Cluster	Closest BLAST Homolog (Organism) Reference (% Identity/% Similarity)	Homologies with Other Siamycin-Like Clusters (% Identity/ % Similarity)			Proposed Function
		*abo* cluster	*msl* cluster	*spe* cluster	
HumA	Aborycin family tricyclic lasso peptide (*Streptomyces* sp. F-7) WP_107308646.1 (95/100)	(92.9/100)	(88.1/97.6)	(86.4/93.2)	Structural gene
HumB1	Lasso peptide biosynthesis PqqD family chaperone (*Streptomyces*) WP_048459499.1 (77/86)	(77/87.4)	(73.6/87.4)	(71.3/86.2)	RiPP recognition element (RRE) protein
HumB2	Lasso peptide biosynthesis B2 protein (*Streptomyces xanthocidicus*) WP_117490375.1 (88/92)	(84.4/90.1)	(86/91.6)	(88.8/91.6)	Leader peptidase
HumC	Lasso peptide isopeptide bond-forming cyclase (*Streptomyces* sp. CB02056) WP_074004732.1 (81/87)	(76.1/84.5)	(76/82.1)	(76.3/84)	Lasso peptide cyclase
HumD1	ATP-binding, cassette domain-containing protein (*Streptomyces yerevanensis*) WP_033320332.1 (86/90)	(73.1/84.7)	(71.4/81)	(78.3/86)	Transporter
HumD2	ABC transporter permease (*Streptomyces scabiei*) WP_037695288.1 (92/94)	(79.6/87.6)	(78.1/88.4)	(72.4/82.4)	Transporter
HumD3	ATP-binding, cassette domain-containing protein (*Streptomyces xanthocidicus*) WP_117490377.1 (83/90)	(81.9/90.3)	(81/88.5)	(84.7/91.3)	Transporter
HumD4	Multispecies: ABC transporter permease subunit (*Streptomyces*) WP_095851496.1 (87/91)	(82.1/89.7)	(85.9/91.8)	(82.1/88)	Transporter
HumE	DoxX family membrane protein (*Streptomyces specialis*) WP_059005885.1 (71/80)	(66.1/75.4)	(66.5/74.9)	(63.8/72.4)	Disulfide oxidoreductase
HumF	Thioredoxin domain-containing protein (*Streptomyces xanthocidicus*) WP_117490379.1 (76/85)	(67.2/76.8)	(70.9/79.9)	(67.9/75.6)	Disulfide oxidoreductase
HumG	Two-component system sensor kinase (*Streptomyces* sp. NL15-2K) GCB43196.1 (79/87)	(72.5/82)	(73.6/81.2)	(69.5/77.7)	Regulator
HumH	Multispecies: CapA family protein (*Streptomyces*) WP_074004738.1 (81/86)	(73.4/83)	(74.1/83.6)	(75.9/83.4)	Putative epimerase
HumR1	Response regulator transcription factor (*Streptomyces* sp. CB02056) WP_079272571.1 (75/86)	(63.1/78.1)	(66.5/79.6)	(71.4/82.3)	Regulator
HumR2	Multispecies: response regulator transcription factor (*Streptomyces*) WP_046710140.1 (93/97)	(84.4/92)	(87.9/93.3)	(84.8/92.9)	Regulator

**Table 2:** Primers used in this study.

Oligonucleotide	Sequence (5’-3’)	Purpose
HumidA_fw	CATGCCGCCCCGTAATTTC	Structural gene amplification
HumidA_rv	GAGAGGTCGGCGCTGATC	Structural gene amplification
pCAPHumGibF	AAAGCTCGTTCTATCGCTTTGCCTCGTTCGTCGAGACTTGAGGTACCTGT	Gibson Assembly
pCAPHumGibR	ATCGCCCAGGTCATCCAGGAGAGCATCGACTCGAGGTTACTAGTCGATCT	Gibson Assembly
HumGib1F	GTCGATGCTCTCCTGGA	Gibson Assembly
HumGib1R	CGAATTCCATGTCGCCTC	Gibson Assembly
HumGib2F	TCTGCTTCTGGTGATCA	Gibson Assembly
HumGib2R	CCTGCTTGACGTTCATC	Gibson Assembly
HumidGib3F	CTACCGCTTCTCCCGTAC	Gibson Assembly
HumidGib3R	AACATCGTCAGGGCCAG	Gibson Assembly
HumidGib4F	TCCGACGAGACGCTGTC	Gibson Assembly
HumidGib4R	CGAACGAGGCAAAGCGAT	Gibson Assembly
